# Interleukin Receptor Associated Kinase 1 Signaling and Its Association with Cardiovascular Diseases

**DOI:** 10.31083/j.rcm2303097

**Published:** 2022-03-12

**Authors:** Youjing Zheng, Jia-Qiang He

**Affiliations:** 1 Department of Biomedical Sciences and Pathobiology, College of Veterinary Medicine, Virginia Tech, Blacksburg, VA 24061, USA

**Keywords:** interleukin-1 receptor associated kinase 1, innate immune response, signaling pathways, toll-like receptor, interleukin-1 receptor, endothelial cells, vascular smooth muscle cells, cardiomyocytes, atherosclerosis, myocardial infarction, heart failure

## Abstract

Toll-like receptors (TLRs) and interleukin-1 receptor (IL-1R) directly interact with intracellular interleukin receptor associated kinase (IRAK) family members to initialize innate immune and inflammatory responses following activation by pathogen-associated or host-derived elements. Although four IRAK family members [IRAK1, 2, 3 (*i.e.,* IRAK-M), and 4] are involved in TLR and IL-1R signaling pathways, IL-1R > IRAK1 signaling appears to be the most studied pathway, with sufficient evidence to support its central role linking the innate immune response to the pathogenesis of various diseases, including cancers, metabolic disorders, and non-infectious immune disorders. However, IRAK1’s involvement in cardiovascular diseases was only recently revealed and the detailed mechanism underling the pathogenesis of cardiovascular diseases, such as atherosclerosis, myocardial infarction, and heart failure (all non-infectious disorders), remains largely unknown with very limited publications to date. This review aims to summarize the overall roles of the IRAK family, especially IRAK1, in mediating the development of cardiovascular diseases.

## 1. Introduction

Cardiovascular disease (CVD) is the leading cause of death worldwide [1]. The mechanisms underlying CVD are extremely complex, involving interactions among multiple local and global factors [2]. Over the past several decades, evidence has demonstrated that both the innate and adaptive immune systems play essential roles in maintaining homeostasis and in the development of CVD [3]. For example, atherosclerosis, originally only referred to as an arterial disease, is now also classified as a chronic inflammatory disease [4]. Similarly, myocardial infarction (MI) was also found to be highly associated with immune responses [5]. Therefore, prevention, reduction, and inhibition of inflammation-induced damage might be an effective approach to prevent and treat CVD in addition to classical medication and surgery.

Traditionally, the immune system can be divided into two categories, the innate immune system and the adaptive immune system [6,7]. The innate immune system is the body’s first defense for both infectious and noninfectious pathogens, where innate immune cells, such as neutrophils, monocytes, macrophages, and dendritic cells, are activated by pathogen-bound toll-like receptor (TLR) signaling [8]. Interestingly, endothelial cells (ECs), which line the inside of the heart and all blood vessels and directly interact with the bloodstream, can also present exogenous antigens to either CD4^+^ or CD8^+^ thymus (T) lymphocytes (*i.e.,* CD4^+^ or CD8^+^ T cells) [9], thus modulating immune responses [10]. The fact that EC dysfunction is a hallmark of multiple CVDs, such as atherosclerosis and aneurysms, reflects their central role in the pathogenesis of immune-associated CVD [11].

As in other tissues and organs in the body, both the innate and adaptive immune systems also protect the cardiovascular system immediately following infection (*e.g.,* virus and bacteria) or non-infection (*e.g.,* MI and heart arrest) injury [12]. The innate immune system is an early responder to cardiac injury and involves activation and recruitment of pro-inflammatory innate immune cells to the damaged site; the intermediate and late stages comprise activation and recruitment of anti-inflammatory immune cells for tissue remodeling and repair [5]. However, both over- and under-reaction (*e.g.,* cytokine storm-induced EC damage in COVID-19 [13] and human cytomegalovirus-induced cardiac dysfunction [14]) of either immune system can lead to CVD [5].

Two molecular patterns are now known to individually or simultaneously activate cardiac resident innate immune cells via pattern recognition receptors (PRRs), such as TLRs and nucleotide-binding oligomerization domain (NOD)-like receptors (NLRs) on stressed ECs [15]. These molecular patterns include (a) Pathogen-associated molecular patterns (PAMPs), such as circulating endotoxin; and (b) Damage-associated molecular patterns (DAMPs), such as cellular debris from injured cardiomyocytes. Among these patterns, the interleukin receptor-associated kinase (IRAK) family plays a crucial role in both TLR- and interleukin-1 receptor (IL-1R)-based activation and modulation of the innate immune response following cardiovascular injury [16].

Well-documented evidence demonstrates that IRAK1 is one of the central kinases involved in the development of various diseases, such as cancer, metabolic disorders (*e.g.,* diabetes), infection (*e.g.,* sepsis), and non-infectious immune diseases (*e.g.,* systemic lupus erythematous) [17]. Because the cardiovascular system (heart and vessels) is known to be modulated by inflammatory cytokines, immune cells, and metabolites [18,19], it is not surprising that IRAK1, as a key mediator of the innate immune response, plays an essential role in the pathogenesis of CVD. Although new evidence shows that other IRAK members (*e.g.,* IRAK3 [16]) are also involved in the development of CVD, we will focus on the overall roles of IRAK1 in modulating inflammatory responses to MI and vascular injury in this review, due to the very limited publications on other IRAK members regarding their association with CVD. Increasing evidence indicates that IRAK1 not only promotes CVD progression but also has the potential to become a new therapeutic target for drug discovery and future treatment of CVD patients.

## 2. IRAK Family Members and IRAK1 Subtype

### 2.1 IRAK Family

The IRAK family is made up of 4 members—IRAK1, IRAK2, IRAK3 (also known as IRAK-M), and IRAK4 [20]. All members share similar functional and structural domains, including a N-terminal death domain (DD), a proline/serine/threonine (ProST) domain, and a kinase or pseudokinease domain (KD or PKD) [21]. Except for IRAK4, the rest of the IRAK members contain an additional C-terminal domain (CD), which is required for activation of tumor necrosis factor receptor-associated factor 6 (TRAF6) [21]. DD plays an important role in TLR and IL-1R signaling by interacting with the myeloid differentiation primary response protein 88 (MyD88). Whereas the ProST domain, which contains 2 peptide sequences rich in proline (P), glutamic acid (E), serine (S), and threonine (T) (the so-called PEST sequence), is responsible for hyperphosphorylation and degradation of IRAK1 [22,23].

IRAKs were first functionally described as key mediators in coordinating multiple IL-1 signaling pathways and in facilitating production of pro-inflammatory cytokines, but later, IRAKs were also found to be implicated in signal transduction through TLRs [21]. Each IRAK family member exerts a different role in modulating TLRs-/IL-1R-associated downstream responses. Following activation of TLRs-/IL-1R, MyD88 and IRAK proteins are recruited to form the receptor complex [22,23], in which IRAK4, as an upstream kinase, activates IRAK1 and IRAK2 through phosphorylation. Phosphorylated IRAK1 and 2 can cause some common or different functional effects. In the early phase, both IRAK1 and IRAK2 have the same function, which is associated with an acute inflammatory response; while in the late phase, IRAK2 is believed to be involved in chronic inflammatory responses [24].

**Fig. 1.**
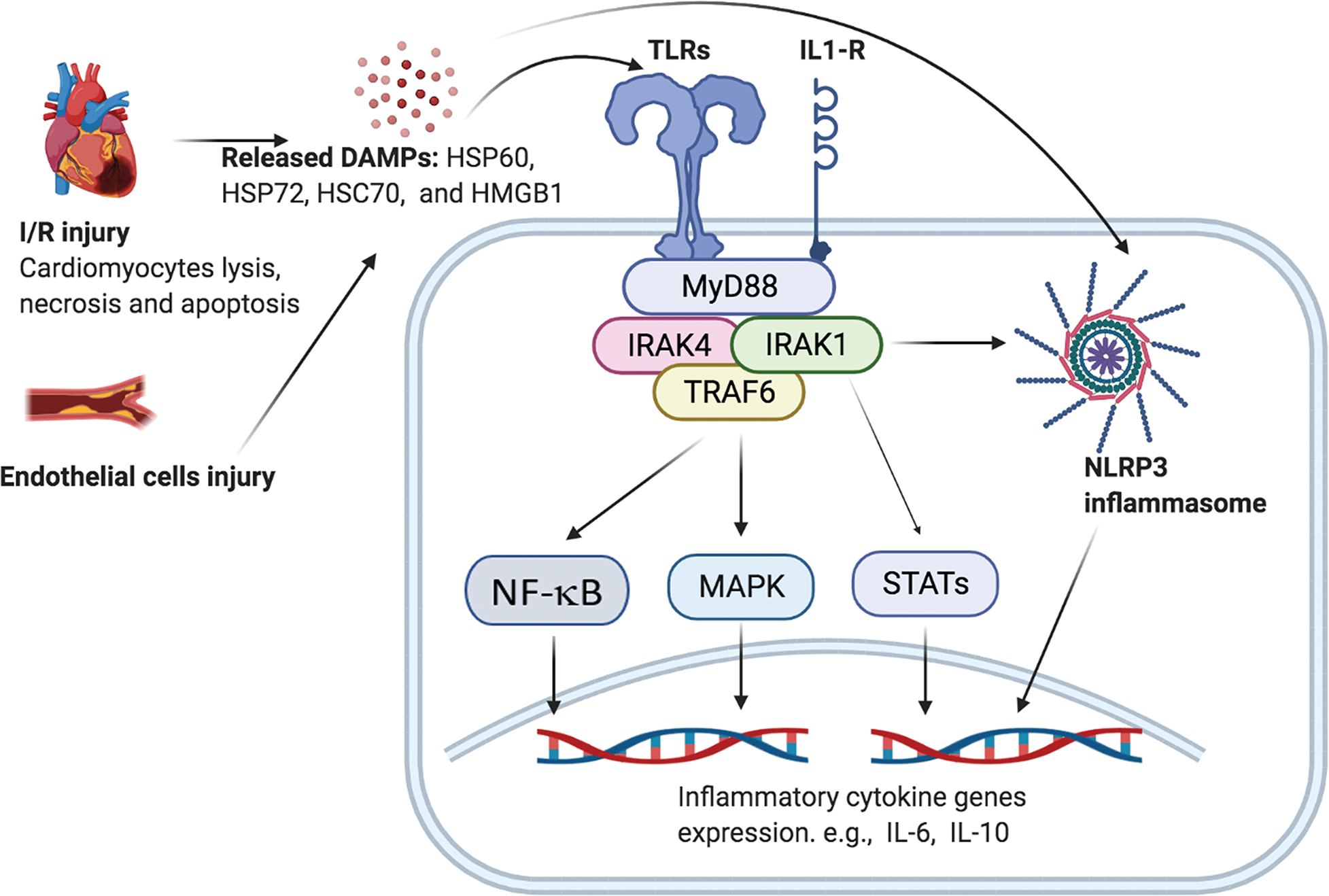
IRAK1-mediated signaling pathways in cardiovascular and immune cells. Injured CMs, ECs, and VSMCs release DAMPs, which then bind to TLRs/IL-1R on immune cells and cardiovascular cells (CMs, ECs, and VSMCs) and activate downstream inflammatory pathways including: (a) IRAK1 > NF-κB and MAPK; (b) IRAK1 > JAK and STAT; and (c) IRAK1 > NLRP3 > Caspase 4/5/11, presumably leading to foam cell formation and cardiac cell apoptosis. The schematic cell refers to an immune (*e.g.,* macrophage) cell or a cardiovascular cell (*e.g.,* CM, ECC, and VSCMC). Abbreviations: DAMPs, damage associated molecular patterns; HMGB1, high mobility group box 1; HSP 60/70/72, heat shock protein 60, 70, 72; I/R, ischemia/reperfusion; IL-1/−6/−10/−18/−27, interleukin-6,−10,−18, −27; IL-1R, interleukin-1 receptor; IRAK1/4, interleukin receptor associate kinase 1/4; MAPK, mitogen-activated protein kinase; MyD88, myeloid differentiation primary response 88; NF-κB, nuclear factor kappa-light-chain-enhancer of activated B cells; NLRP3, nucleotide-binding domain, leucine-rich-containing family, pyrin domain-containing-3; STAT, signal transducers and activators of transcription; TLRs, toll-like-receptors; TRAF6, tumor necrosis factor receptor-associated factor 6 (The figure was originally created by the authors using BioRender.com online software).

Activation of IRAKs (1, 2, and 4) by TLRs/IL-1R leads to stimulation of various downstream signaling pathways, especially the nuclear factor kappa B (NF-κB)/mitogen-activated protein kinase (MAPK) pathway (NF-κB/MAPK) [25], the Janus kinase (JAK)/signal transducer and activator of transcription (STAT) pathway (JAK/STAT) [26], and the NLR protein 3 (NLRP3)/Caspase-4/−5/−11 pathway (NLRP3/Caspase-4/−5/−11) [18], which eventually stimulates production and secretion of pro-inflammatory cytokines (Fig. 1). In contrast, IRAK3 negatively modulates TLRs/IL-1R (or TIR) domain signaling by inhibiting the IRAK1–2-4 complex, reducing production and secretion of inflammatory cytokines, eventually resulting in immunosuppression [27,28].

### 2.2 IRAK1 Subtype

IRAK1, the first-discovered and most-studied member of the IRAK family, is known to play an important role in mediating IL-1-associated immune and inflammatory responses by activating NF-κB pathway [29]. It was found that human IRAK1 is ubiquitously expressed in almost every organ, whereas mouse IRAK1 appears to be manifested mainly in the liver, kidneys, and testis [29].

IRAK1 comprises 3 splice variants (1b, 1c, and 1s) as well as a full-length form and each variant has its unique biological function. For example, IRAK1b remains active and extremely stable after being activated by IL-1 signaling, thus, leading to the sustainable activity of NF-κB [30]. IRAK1c, which is functionally similar to IRAK3, is a dominant-negative regulator in TIR domain-mediated immune and inflammatory responses [31]. IRAK1c is dominantly expressed in human brain and may be mainly involved in neuronal responses to infection and tissue damage [30,32]. Lastly, IRAK1s appears to be expressed only in murine mammals as an inactive kinase. Although its biological function remains unclear, overexpression of *IRAK1s* stimulates the downstream NF-κB and c-Jun N-terminal kinase (JNK) pathway via binding to endogenous IRAK1[33]. Nevertheless, existence of IRAK1 splice variants with different biological functions and capacities demonstrate the diverse impacts of the IRAK1 subfamily on innate immune and inflammatory responses. The different expression of *IRAK1* variants between mice and humans may signify their common and also special requirements for immune and inflammatory responses between the two species [34], which requires further investigation.

## 3. IRAK1 Signaling and Its Association with Cardiovascular Diseases

### 3.1 TIR Domain-associated IRAK1 > JAK > STAT and IRAK1 > NLRP3 > Caspase Signaling in Non-cardiovascular Cells Are Presumably Similar to Those in Cardiovascular Cells

The involvement of TIR domain-associated IRAK1 > JAK > STAT and IRAK1 > NLRP3 > Caspase signaling in cardiac cells is largely unknown and published studies using cardiovascular cells are very limited. Although most of the published data in this area is in non-cardiac cells, such as macrophages, lymphocytes, and microglial cells (Fig. 1) [35,36], the findings are likely similar in cardiovascular cells. Huang *et al.* [37] reported that IRAK1 is essential for STAT3 activation and subsequent expression of interleukin-6 and 10 (IL-6, IL-10) because IRAK1-deficient cells showed impaired IL-10 production. It is possible that IRAK1 directly binds to the IL-10 promoter upon lipopolysaccharide (LPS) challenge or modulates STAT3 activity via TRAF6, increasing production of inflammatory cytokines [38]. IRAK1 also participates in STAT1 activation in IL-1R signaling [38]. In IRAK1-deficient macrophages, higher levels of STAT1/2 activities and IL27 production increase following IFN-β stimulation [39]. Since IL-10 and IL-27 are anti-inflammatory cytokines, IRAK1-mediated IL-1R to STAT signaling may play a role in the late stage tissue repair and remodeling, while IRAK1-mediated production of pro-inflammatory cytokines in NF-κB/MAPK signaling may engage in the early stage of infection and inflammation [35,36].

NLRs are the only cytoplasmic receptors that recognize pathogen-derived intracellular invaders (*i.e.,* PAMPs) and non-infectious danger signals (*i.e.,* DAMPs from injured cardiac cells [40]) among the four members of PRR [TLRs, NLRs, C-type lectin receptors (CLRs), and RIG-1 like receptors (RLR)] [41,42] (also see Fig. 1). Activation of NLRs by TIR domain signaling promotes the formation of inflammasomes, which then activate Caspase-1/4/5/11, leading to the production of the pro-inflammatory cytokines IL-1β, and IL-18 [40,42]. In this signaling response, NLRP3 appears to be the most important factor and involves a wide range of inflammation and autoimmune-induced disorders in patients [40]. Emerging evidence indicates that IRAK1 plays an essential role in the rapid activation of NLRP3, thus, promoting TIR domain-associated immune responses [41].

### 3.2 TIR Domain-associated IRAK1 > NF-κB and MAPK Signaling Pathways in Non-cardiovascular and Cardiovascular Cells

The first known role of IRAK1 is the mediation of signal transduction from TLRs to inflammatory cytokines via the NF-κB and MAPK pathways (Fig. 1). TLRs belongs to the PRR family and are Type I single-spanning glycoprotein [24]. TLRs are mainly expressed in immune cells [*e.g.,* macrophages, mast cells, dendritic cells, nature killer (NK) cells, T cells, and B cells], but they were also identified in cardiomyocytes, epithelial cells, ECs, and vascular smooth muscle cells (VSMCs) [43]. As to cellular distribution, TLR1–2, TLR4–6, and TLR11 are found on the cell surface while TLR3 and TLR7–9 are located on the membranes of intracellular organelles, such as endosomes and lysosomes [44]. In the heart of mouse and human, TLR4 is probably the most abundant TLR among all 13 TLR family members [45] and it is the only LPS receptor that is associated with an immediate inflammatory response in heart following myocardial ischemia and sepsis [3], implying its unique biological role in modulating cardiac responses to both infection- and non-infection-induced injury.

TLRs can be recognized and activated by two types of ligands, PAMPs and DAMPs as mentioned above. PAMPs refer to infectious pathogens, such as PS, phospholipids, extracellular matrix, peptide, and nucleic acids released from bacteria; while DAMPs denote non-infectious endogenous molecules, such as heat shock proteins 60/70/72 (HSP60, 70, and 72) and high mobility group box 1 (HMGB1) protein released from, for example, injured cardiomyocytes [3,46]. Studies indicate that all TLRs contain an extracellular domain with leucine-rich repeat (LRR) motifs specific for recognizing TLR ligands, whereas all IL-1Rs contain three immunoglobulin-like domains (Ig-like domains) specific for recognizing IL-1 ligands [24]. Since TLRs and IL-1Rs share the same homologous TIR domain, IRAK1 could participate in both TLR- and IL-1R-mediated signaling, suggesting its biological importance [24].

Activation of TLRs/IL-1R causes a series of intracellular reactions in the downstream signaling cascades, including recruitment of the TIR domain adaptors such as MyD88 [47], formation of the IRAK1/4 and MyD88 complexes, IRAK1 phosphorylation by IRAK4, detachment of hyper-phosphorylated IRAK1 [17], activation of TRAF6 [48]/transforming growth factor-β-activated kinase 1 (TAK1), phosphorylation/degradation of inhibitory KappaB (I kappa B or IκB) protein by IκB kinases (IKKs), and the nuclear translocation of NF-κB. The final consequence of the above steps will ultimately lead to transcription and production of pro-inflammatory cytokines such as IL-1β, IL-6, and tumor necrosis factor α (TNFα) [48]. Alongside, activation of TLRs/IL-1R also stimulates the MAPK signaling via TAK1 pathway [49]. TAK1 then leads to the phosphorylation of JNK, p38, and, ultimately, cyclic adenosine monophosphate (cAMP) response element-binding protein (CREB), ending up with the activation of activator protein 1 (AP-1). The AP-1 transcription factor thereafter elicits transcription and production of pro-inflammatory cytokines similar to NF-κB [50] (also see Fig. 1).

### 3.3 IRAK1-mediated pathogenesis of atherosclerosis

Atherosclerosis (often denoted as arteriosclerosis), which is characterized by lipid-laden foam cell accumulation in the arterial wall, is recognized as a chronic inflammatory disease [51]. As stated above, activation of IRAK1 enhances production of IL-10 [37] and patients with atherosclerosis often have an elevated level of IL-10, implying that the IRAK1-mediated innate immune response may play an important role in the development of atherosclerosis [37]. Numerous *in vitro* and *in vivo* studies listed below support these findings.

By genotyping 4 loci on the *IRAK1* gene on the X chromosome of 996 Caucasian patients with Type 2 diabetes (467 men and 529 women), the Diabetes Heart Study identified two major haplotypes, CTTT (82%) and TCCG (13%), in the *IRAK1* gene. The TCCG haplotype is significantly correlated with an enhanced blood level of C-reactive protein (CRP, an acute inflammation marker) in women but not in men [52]. Since blood concentration of CRP has been used to assess the risk of coronary artery disease and predict MI and stroke [53], the presence or overexpression of the TCCG haplotype in the *IRAK1* gene may promote pathogenesis of CVDs [52].

**Fig. 2.**
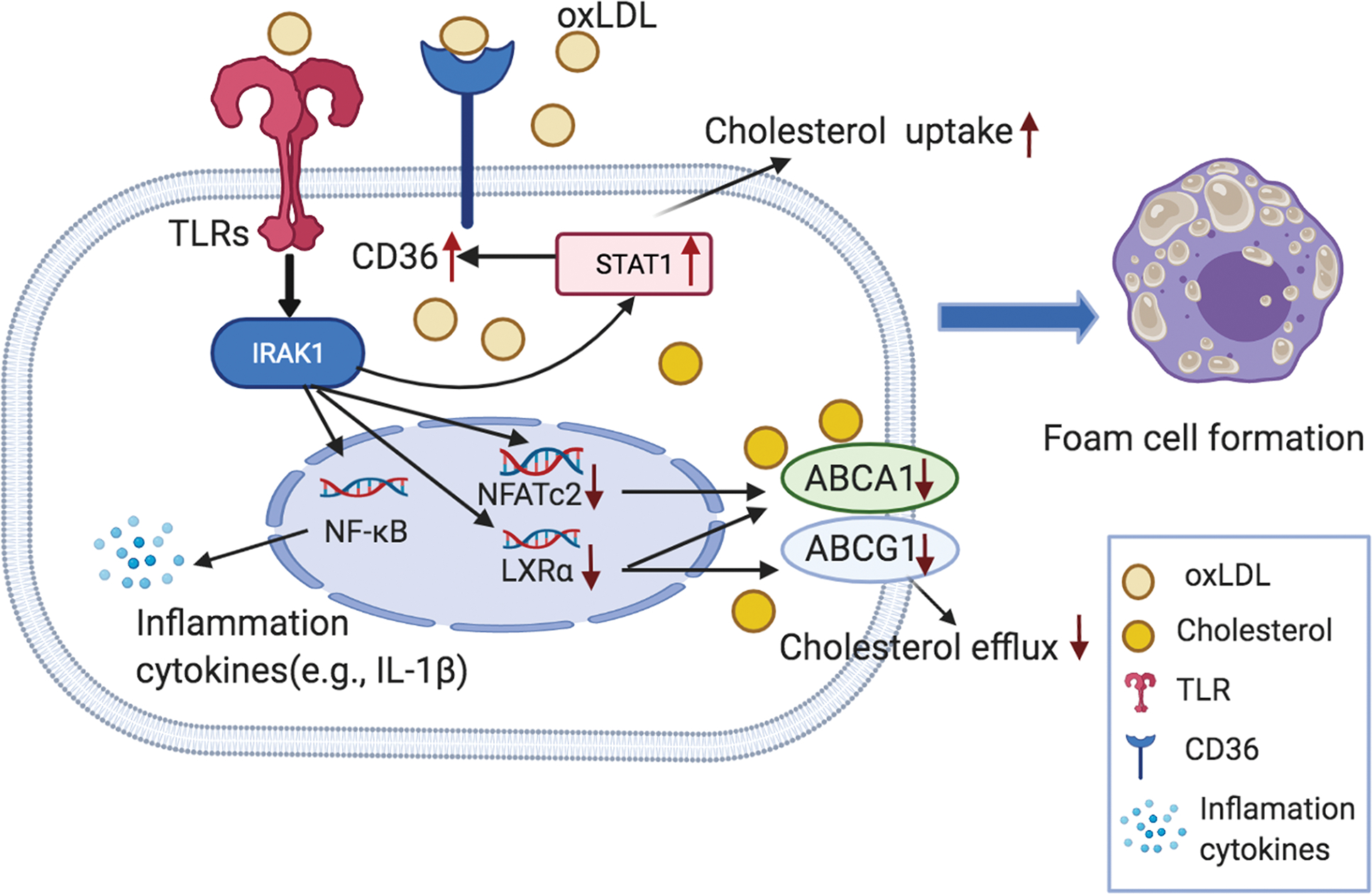
Working hypothesis of IRAK1-induced formation of atheromatous foam cells in the vascular wall. It is known that foam cells are formed from monocyte-derived M_2_ macrophages (majority), synthetic VSMCs, or injured ECs. Once oxLDL binds to TLRs and CD36 receptors on macrophages, ECs, and VSMCs, it will activate the (a) IRAK1 > STAT1; (b) IRAK1 > NF-κB inflammatory; and (c) IRAK1 > NFATc2 and LXRα signaling pathways; together, they promote secretion of pro-inflammatory cytokines and cholesterol efflux and facilitate the formation of foam cells. Abbreviations: ABCA1 (ABCG1), ATP-binding cassette subfamily A (G) member 1; CD36, cluster of differentiation 36; IRAK1, interleukin receptor associate kinase 1; LXRα, liver transcriptional X receptor alpha; NF-κB, nuclear factor kappa-light-chain-enhancer of activated B cells; NFATc2, nuclear factor of activated T cell 2; oxLDL, oxidized low-density lipoprotein; STAT1, signal transducers and activators of transcription 1; TLRs, toll-like-receptors (The figure was originally created by the authors using BioRender.com online software).

On the other hand, the ATP-binding cassette subfamily A member 1 (ABCA1), a crucial mediator of lipid efflux in the cell membrane, can be downregulated by IRAK1 through TLR4 signaling pathway following treatment of oxidized low-density lipoprotein (oxLDL) [54], suggesting IRAK1’s potential role in atherosclerotic development. This hypothesis is supported by a similar study, in which IRAK1 was found to increase lipid binding, uptake, and cholesterol efflux in foam cells [55] (also see Fig. 2). However, inhibition of IRAK1 (by an IRAK1 antagonist, such as IRAK1/4 inhibitor or its siRNA) significantly attenuated expression of cluster differentiation 36 (CD36) [55]. It is known that CD36 is a crucial macrophage scavenger receptor that is responsible for cellular cholesterol accumulation, oxLDL internalization, and foam cell formation [55]. Downregulation of CD36 may lead to beneficial effects in preventing or weakening the development of atherosclerosis. Furthermore, inhibition of IRAK1 also increases expression of ABCA1 and ATP-binding cassette subfamily G member 1 (ABCG1), which leads to increased cholesterol efflux from macrophages [55]. This process is accompanied by increased expression and activity of liver transcriptional X receptor alpha (LXRα) and nuclear factor of activated T cell (NFATc2). LXRα serves as a cholesterol sensor and regulates ABCA1 and ABCG1 through NFATc2 [55]. IRAK1 plays a critical role in maintaining NFAT in a phosphorylated inactive state [56]. Suppression of IRAK1 in macrophages enhances binding of NFATc2 to the ABCA1 promoter and results in an increase of ABCA1 expression and cholesterol efflux [57].

IRAK1 also stimulates VSMC proliferation, which is a pivotal pathogenic process in the development of atherosclerosis [58]. Two signaling pathways are likely involved in IRAK1-associated VSMC proliferation: (a) the IL-1/IL-18 inflammatory pathway, in which activation of IRAK1 induces production of inflammatory cytokines such as IL-1 and IL-18 which, in turn, provokes VSMC proliferation during atherosclerosis progression [59]; and (b) the kinase pathway, in which IRAK1 facilitates VSMC proliferation and neointimal hyperplasia by activating protein kinase C (PKC) and extracellular signal regulated kinase (ERK) [60].

ECs, a major cell type involved in plaque formation in atherosclerosis, is also found to be regulated by IRAK1 [61]. Alfaidi *et al.* [62] revealed that a signaling adaptor, named non-catalytic region of tyrosine kinase 1 (Nck1), could interact with IRAK1 under shear stress and trigger NF-κB-based secretion of proinflammatory cytokines. IRAK1 knockdown in ECs inhibits expression of NF-κB and downstream endothelial adhesion molecules, such as vascular cell adhesion molecule-1 (VCAM-1) and intercellular cell adhesion molecule-1 (ICAM-1). These molecules are crucial factors for endothelial activation [63]. Another study found that carotid ligation (disturbing shear stress) in mice increases phosphorylation of IRAK1 and expression of Nck1 in ECs, supporting the human findings in which both Nck1 and IRAK1 expression levels are increased in ECs isolated from human carotid artery plaques [62]. Correlations among constitutive expression of IRAK1 in the peripheral blood mononuclear cells isolated from patients with atherosclerosis, elevated levels of the plasma IL-10, and the stimulation effect of IRAK1 on IL-1 [37,64], imply direct involvement of IRAK1 in atherosclerotic development. Collectively, these data establish a link between disturbed blood flow and IRAK1-mediated inflammation, highlighting a unique role for IRAK1 as a specific Nck1 binding protein in mediating endothelial activation under atheroprone hemodynamics [62].

### 3.4 IRAK1-mediated Pathogenesis of Myocardial Infarction (MI) and Heart Failure (HF) via IRAK1 or miRNA > IRAK1 Pathway

Growing evidence demonstrates that IRAK1 is also involved in the development of cardiac diseases, such as MI and HF (Table 1, see Reference [65–69]). Thomas *et al.* [65] reported that IRAK1 mediates LPS-induced myocardial dysfunction of contractile through TIR domain signaling and knockout of the *Irak1* gene significantly reduces mortality of mice with HF. Because atherosclerotic-induced MI and bacterial-induced cardiac septic shock share common characteristics of myocardial dysfunction, pharmacological inhibition of IRAK1 may reduce cardiac inflammation and provide beneficial effects for patients with heart disease [3]. Similarly, studies using a mouse ischemia/reperfusion model found that TLR4 > IRAK1 signaling is activated and HSP60 expression is increased in injured cardiomyocytes. Although the underlying mechanism is not well-known, it is probably related to activated signaling from HSP60 (released from injured cardiomyocytes) > TLR2/4 > IRAK1 > apoptosis in cardiomyocytes [66,70].

Recent studies suggest that small non-coding RNAs [*e.g.,* microRNA (miRNA), small nuclear RNA (snRNA), small nucleolar RNA (snoRNA), and Piwi-interacting RNA (piRNA)] are also involved in pathogenesis through modulation of IRAK1 [71]. Among ~2000 miRNAs, miRNA-146a was first identified as a negative regulator targeting IRAK1 and TRAF6 and reduced the TLR-triggered innate immune response [72]. This finding was supported by a gain-of-function study using the same model of mouse MI. In this study, increased expression of miRNA-146a decreased cardiac infarct size (~50%) and increased cardiac function, which was believed to be due to direct inhibition of IRAK1 and TRAF6 by miRNA-146a [68]. Another study also found that miRNA-146a diminishes sepsis-induced cardiomyocyte apoptosis and infiltration of inflammatory monocyte cells by inhibiting the same molecule (*i.e.,* IRAK1 and TRAF6) [67]. New studies found that IRAK1 can also be regulated by other miRNA, such as miRNA-142–3p. Su *et al.* discovered that there is significant downregulation of miRNA-142–3p in porcine MI induced by coronary micro-embolization. Either up-regulation of miRNA-142–3p or downregulation of IRAK1 appears to be able to reduce production of NF-κB and pro-inflammation cytokines (IL-1β, IL-6) in cardiomyocytes [69], which could become potential pharmaceutical targets to treat patients with MI.

## 4. Conclusions

IRAK1 is the most studied IRAK member within the TLR and IL-1R signaling pathways, where it plays an essential role initializing the innate immune response to both infectious pathogen invasion and non-infectious injuries. Much of the collected data has indicated that IRAK1 is highly associated with the pathogenesis of CVD, especially in the development of atherosclerosis, MI, and HF. Overall, this review demonstrates that IRAK1 is not only activated by multiple signaling molecules (*e.g.,* TLR/IL-1R ligands) but it can also be inhibited by pharmacological agents with beneficial results, suggesting its potential as a new therapeutic target (in addition to other known targets, such as IL-1 and NLRP3) for drug discovery and development for patients with CVD. However, the detailed mechanisms underlying IRAK1-promoted cardiovascular injury, remodeling, and regeneration remain largely unknown. For example, what is the specific role of IRAK1 in the innate immune cells in response to MI following I/R? Are there any differences between cardiomyocytes and immune cells in terms of the function of IRAK1? How can the protective efficacy of the commonly used pre-conditioning strategy be enhanced by modulating IRAK1-mediated signaling? Is IRAK1 a better therapeutic target than other components (*e.g.,* MyD88, IRAK4) within the TLR pathway? A substantial amount of work remains to be conducted to answer these questions.

## Figures and Tables

**Table 1.** A list of published animal studies that focus on IRAK1 signaling-associated cardiac diseases.

Cardiac Disease	Animal Model	Experimental target	Major Findings and Conclusion

Septic HF [65]	IRAK1^−/−^ transgenic mice; LPS-induced cardiac disorder	IRAK1	➢ Impaired TLR/IL-1R signaling transduction ➢ Resists acute LPS-induced contractile dysfunction ➢ Prolonged lifespan with severe myocarditis and lethal HF for 150 days
MI (I/R) [66]	TLR4^−/−^ and MyD88^−/−^ double knockout mice; LAD ligation-induced MI	TLR4 and MyD88	➢ MI-induced IRAK1 activation is TLR4- and MyD88-dependent via HSP60 ➢ HSP60 significantly activated Caspase-3 and -8 in the FADD pathway
Septic HF [67]	LPS-induced cardiac disorder in mice	miRNA-146a	➢ Transfection of miRNA-146a attenuated LPS-induced cardiac dysfunction and infiltration of inflammatory cells in the heart tissue
MI (I/R) [68]	LAD ligation-induced MI in mice	miRNA-146a	➢ Transfection of miRNA-146a decreased infarct size and increased EF% of MI mice via suppressing IRAK1/TRAF6 signaling
MI (I) [69]	CME-induced MI in pig	miRNA-142	➢ CME downregulates miRNA-142-3p and leads to HF ➢ AgomiR and antagomiR exert opposite effects

**Abbreviations:** CM: cardiomyocytes; CME: coronary microembolization; EF: ejection fraction; FADD: FADD: fas-associated death domain protein; HF: heart failure; HSP60: heat shock protein 60; I/R: ischemia-reperfusion; IRAK1: interleukin-1 receptor-associated kinase 1; IL-1R: interleukin-1 receptor; LAD: left anterior descending; LPS: lipopolysaccharides; MI: myocardial infarction; miRNA: microRNA; MyD88: myeloid differentiation primary response protein 88; NF-κB: nuclear factor kappa-light-chain-enhancer of activated B cells; TLR4: toll-like receptor 4; TRAF6: tumor necrosis factor receptor-associated factor 6.
